# 

**Published:** 1880-09

**Authors:** 


### CONTENTS.

COMMUNICATIONS.

Relations of the Profession and the Public.......... 381
Treatment of Deciduous Teeth.......... 390
Proceedings of the Twentieth Annual Meeting of the American Dental Association.......... 395
Correspondence.......... 420

EDITORIAL.

The American Dental Association.......... 420
"In Chains".......... 421
Bibliographical.......... 423
Obituary.......... 423
Obituary.......... 424

